# Metric scale non-fixed obstacles distance estimation using a 3D map and a monocular camera

**DOI:** 10.3389/frobt.2025.1560342

**Published:** 2025-06-12

**Authors:** Daijiro Higashi, Naoki Fukuta, Tsuyoshi Tasaki

**Affiliations:** Graduate School of Science and Technology, Meijo University, Nagoya, Japan

**Keywords:** obstacle detection, depth completion, monocular depth estimation, 3D map, semantic segmentation, autonomous driving

## Abstract

Obstacle avoidance is important for autonomous driving. Metric scale obstacle detection using a monocular camera for obstacle avoidance has been studied. In this study, metric scale obstacle detection means detecting obstacles and measuring the distance to them with a metric scale. We have already developed PMOD-Net, which realizes metric scale obstacle detection by using a monocular camera and a 3D map for autonomous driving. However, PMOD-Net’s distance error of non-fixed obstacles that do not exist on the 3D map is large. Accordingly, this study deals with the problem of improving distance estimation of non-fixed obstacles for obstacle avoidance. To solve the problem, we focused on the fact that PMOD-Net simultaneously performed object detection and distance estimation. We have developed a new loss function called “DifSeg.” DifSeg is calculated from the distance estimation results on the non-fixed obstacle region, which is defined based on the object detection results. Therefore, DifSeg makes PMOD-Net focus on non-fixed obstacles during training. We evaluated the effect of DifSeg by using CARLA simulator, KITTI, and an original indoor dataset. The evaluation results showed that the distance estimation accuracy was improved on all datasets. Especially in the case of KITTI, the distance estimation error of our method was 2.42 m, which was 2.14 m less than that of the latest monocular depth estimation method.

## 1 Introduction

Accurate obstacle distance estimation is important for ensuring the safety of autonomous driving cars and mobile robots. Light detection and ranging (LiDAR) has been conventionally used for measuring distance with a metric scale, but LiDAR is expensive. Hence, this study aims to achieve metric scale distance estimation using an inexpensive monocular camera. The other comparison items are shown in Table 1. Here, we compare a camera with a 360-degree mechanical 3D LiDAR that is used generally. The merits of LiDAR are the accuracy and 360-degree field of view. However, the durability is low because of its mechanical rotation parts. The merits of a camera are the cost, resolution, frame rate, and ability of getting color information.

**TABLE 1:** Comparison between a LiDAR and a camera.

Properties	LiDAR	Camera
Accuracy	High	Normal
Cost	Very expensive	Inexpensive
Field of view	360 deg	About 70 deg–150 deg
Resolution	Low	High
Durability	Normal	High
Frame rate	Normal	High
Other feature		Get color information

**FIGURE 1:**
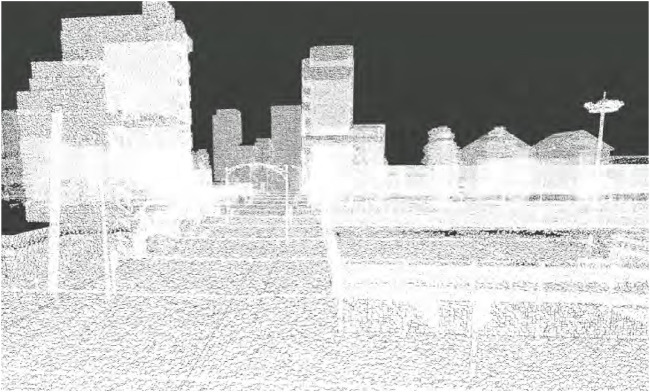
Example of the 3D map used in PMOD-Net. In this study, a point-cloud-based 3D map is assumed.

Highly accurate depth estimation models using a monocular camera are NDDepth (Shuwei et al., 2024) and IEBins (Shuwei et al., 2023). However, the depth estimated by these methods has no scales in the place where they are not trained. We have developed PMOD-Net (Shikishima et al., 2023), which accurately estimates distance with a metric scale by using a monocular camera and a 3D map, as shown in Figure 1. PMOD-Net performs semantic segmentation and distance estimation simultaneously from a depth image created by a 3D map and a camera image. PMOD-Net’s semantic segmentation contributes to the detection of non-fixed obstacles that do not exist on the 3D map. In this study, non-fixed obstacles are defined as all obstacles that do not exist on the map. Therefore, not only moving objects but also static objects such as parked vehicles are included in the non-fixed obstacles. However, the accuracy of distance estimation is still low because the 3D map has no information of the distance to the non-fixed obstacles. In this study, we address the novel challenge of improving the accuracy of distance estimation for non-fixed obstacles that do not exist on the 3D map.

To address this challenge, we focused on the semantic segmentation results provided by PMOD-Net. We develop a new loss function paying attention to the regions detected as non-fixed obstacles by PMOD-Net. This approach is the first attempt to specialize in training a neural network to measure the distance to non-fixed obstacles that do not exist on the 3D map.

To summarize, the contributions of this work are listed as follows:

• A new loss function is developed to improve the accuracy of the world’s first neural network for distance estimation using a 3D map and a monocular camera.
•We achieve higher accuracy on the public dataset KITTI-360 (Liao et al., 2022) compared to the latest off-the-shelf monocular depth estimation method.
•We verify that our method works well on a mobile robot that has a camera.

## 2 Related studies

### 2.1 Depth completion with a metric scale

There are many depth completion methods that utilize LiDAR point cloud and a monocular camera image as input (Sindagi et al., 2019; Wang et al., 2021; He et al., 2019; Hai et al., 2023; Guo et al., 2022). These methods can complement the distance with a metric scale because the distance is complemented by the 2D image and the LiDAR point cloud, which has a metric scale. In the case of regarding a 3D map as a pseudo LiDAR point cloud, it is possible to reconstruct depth image from the 3D map and monocular camera image. However, depth completion methods require the synchronization of the LiDAR point cloud and 2D image. That is, all obstacles that exist in an image must also be in the point cloud. Therefore, even when 3D map is adapted to the depth completion methods, they do not provide as accurate distance estimation as PMOD-Net (Shikishima et al., 2023)

### 2.2 Monocular depth estimation

Monocular depth estimation is used in a lot of technical fields, including robotics (Jia et al., 2023) and augmented reality (Lee et al., 2011). MIMDepth (Xie et al., 2022), based on GLPDepth (Kim et al., 2022), significantly contributes to monocular depth estimation in the field of autonomous driving. NDDepth performs better than the previous methods on the KITTI (Geiger et al., 2012) and NYUDepth-v2 (Silberman and Derek Hoiem Pushmeet Kuhli, 2012) datasets. NDDepth realizes an accurate estimation by introducing a normal-distance head in addition to planar detection through Felzenszwalb segmentation (Felzenszwalb and Huttenlocher, 2004).

Zero-shot estimation is a challenge in the field of monocular depth estimation, which requires large amounts of training data. Recent models such as Depth Anything (Yang et al., 2024) have been developed to provide highly accurate estimation in a zero-shot setting.

Many monocular depth estimation models have been developed. However, scale estimation is required when estimating in an environment different from the one in which the model was trained. As a result, the accuracy of scale estimation can significantly affect the overall accuracy of the model. PMOD-Net (Shikishima et al., 2023) has no problems of scale estimation because it uses a 3D map as an input.

### 2.3 PMOD-Net

**FIGURE 2:**
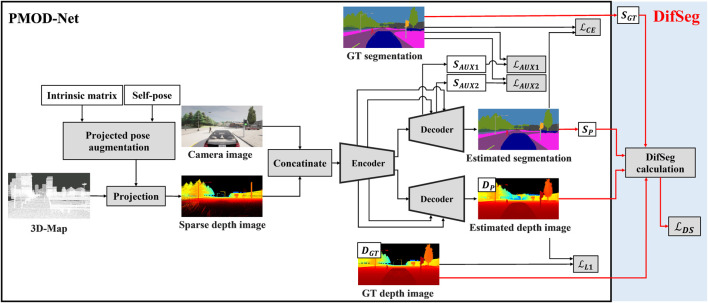
Architecture of PMOD-Net with DifSeg loss. The black frame shows the architecture of the conventional PMOD-Net.

PMOD-Net (Shikishima et al., 2023) is the world’s first neural network for metric scale obstacle detection using a 3D map and a monocular camera. Figure 2 shows the architecture of PMOD-Net within the black frame. The inputs are a sparse depth image projected from a 3D map and a camera image from a monocular camera. A sparse depth image is created by projecting a 3D map based on the self-pose on a 3D map. PMOD-Net performs semantic segmentation and distance estimation simultaneously.

PMOD-Net enables distance estimation with a metric scale for non-fixed obstacles that do not exist on the 3D map, thanks to a multitask learning. That is, semantic segmentation detects non-fixed obstacles, which enhances distance estimation for non-fixed obstacles. However, during training, only L1 norm loss ${£}_{L1}$ is used across the entire depth image between the estimated depth image and ground truth (GT) depth image. Therefore, the distant non-fixed obstacles are often not detected, which is a problem. In this paper, to solve the problem, we have developed a new loss function.

## 3 Materials and methods

### 3.1 DifSeg loss

In order to improve the PMOD-Net distance estimation, we propose an additional loss. This additional loss is calculated based on the PMOD-Net estimated and GT segmentation image. We named this additional loss DifSeg (difference of distance based on semantic segmentation).

**FIGURE 3:**
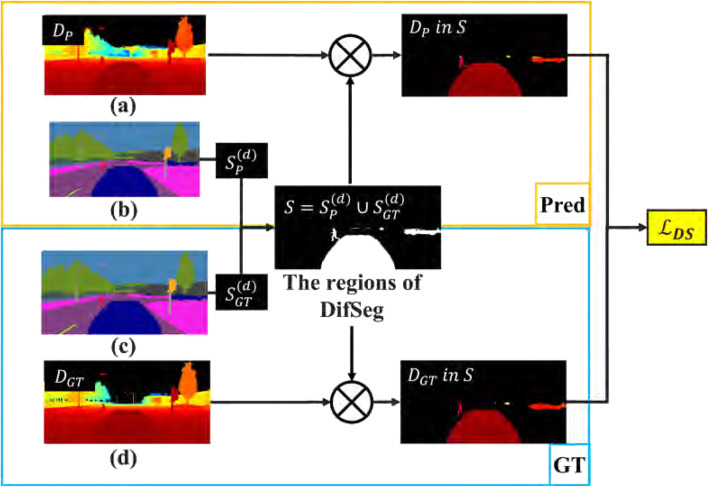
Overview of DifSeg loss. **(a)** Depth image estimated by PMOD-Net. **(b)** Segmentation image estimated by PMOD-Net. **(c)** Ground truth segmentation image. **(d)** Ground truth depth image.

Figure 3 shows the data to calculate DifSeg loss. Figures 3a, b represent a depth image $D_{P}$ and a segmentation image estimated by PMOD-Net during training, respectively. Figures 3c, d represent the GT segmentation image and depth image $D_{GT}$ . The regions identified as non-fixed obstacles in Figures 3b, c denote $S_{P}^{\left(d\right)}$ and $S_{GT}^{\left(d\right)}$ , respectively.

The DifSeg loss is calculated in the region S shown in Equation 1.

$$S=S_{p}^{\left(d\right)}\cup S_{GT}^{\left(d\right)}.$$ (1)

The DifSeg loss ${£}_{DS}$ is defined by Equation 2. Here, j denotes the position of pixels in the region S , and N denotes the number of pixels in the region S .

$${£}_{DS}=\frac{1}{N}\overset{N}{\underset{j=1}{\sum }}|D_{p}\left(j\right)-D_{GT}\left(j\right)|.$$ (2)

The pseudocode to calculate DifSeg is shown in Algorithm 1.

Algorithm 1 Calculate DifSeg.

**Require:** $S_{p}^{\left(d\right)},S_{GT}^{\left(d\right)}$ : binary array
**Require:** $D_{p},D_{GT}$ : float array
**Ensure:** ${£}_{DS}$

  1: $S\leftarrow S_{p}^{\left(d\right)}$ or $S_{GT}^{\left(d\right)}$

  2: $D_{p}^{\left(d\right)}\leftarrow D_{p}\times S$

  3: $D_{GT}^{\left(d\right)}\leftarrow D_{GT}\times S$

  4: N←0

  5: ${£}_{DS}\leftarrow 0$

  6: **for** j←1,2,… , S.length **do**
  7:   **if** S[j]=1 **then**
  8: N←N+1

  9: ${£}_{DS}\leftarrow {£}_{DS}+|D_{p}^{\left(d\right)}\left[j\right]-D_{GT}^{\left(d\right)}\left[j\right]|$

  10:   **end if**
  11:  **end for**
  12:  **return** ${£}_{DS}/N$

PMOD-Net can be trained to focus on non-fixed obstacles by adopting DifSeg loss. Consequently, the accuracy of distance estimation for non-fixed obstacles that do not exist on the 3D map can be improved. Furthermore, by leveraging both GT and estimated segmentation results, strong training of misdetection regions associated with non-fixed obstacles is realized.

### 3.2 Implementation in PMOD-Net

Figure 2 shows the architecture of PMOD-Net with DifSeg loss. As shown in Figure 2, conventional PMOD-Net uses the loss for semantic segmentation ( ${£}_{CE}$ , ${£}_{\mathrm{AUX1}}$ , and ${£}_{\mathrm{AUX2}}$ ) and the loss ${£}_{L1}$ for distance estimation. ${£}_{\mathrm{AUX1}}$ and ${£}_{\mathrm{AUX2}}$ and ${£}_{CE}$ are cross-entropy losses for semantic segmentation. $S_{p}$ denotes the final output from the decoder. $S_{\mathrm{AUX1}}$ and $S_{\mathrm{AUX2}}$ are up-sampled outputs branched just before the skip connection from the encoder. $S_{p}$ , $S_{\mathrm{AUX1}}$, and $S_{\mathrm{AUX2}}$ are used for calculating loss, as shown in Equations 3–5, respectively. Here, n denotes the number of pixels of the output segmentation image.

$${£}_{CE}=-\overset{n}{\underset{i=1}{\sum }}\left(S_{GT}\left(i\right)\log\left(S_{p}\left(i\right)\right)\right).$$ (3)

$${£}_{\mathrm{AUX1}}=-\overset{n}{\underset{i=1}{\sum }}\left(S_{GT}\left(i\right)\log\left(S_{\mathrm{AUX1}}\left(i\right)\right)\right).$$ (4)

$${£}_{\mathrm{AUX2}}=-\overset{n}{\underset{i=1}{\sum }}\left(S_{GT}\left(i\right)\log\left(S_{\mathrm{AUX2}}\left(i\right)\right)\right).$$ (5)

${£}_{L1}$ represents the L1 loss for distance estimation, comparing the final output from the decoder with the GT depth image. ${£}_{L1}$ is defined by Equation 6.

$${£}_{L1}=\frac{1}{n}\overset{n}{\underset{i=1}{\sum }}\left|D_{p}\left(i\right)-D_{GT}\left(i\right)\right|.$$ (6)

Our new PMOD-Net with DifSeg is trained by using loss £ defined by Equation 7. The $\lambda_{1}$ , $\lambda_{2}$ , $\lambda_{3}$ , $\lambda_{4}$, and $\lambda_{5}$ denote weight parameters.

$$£=\lambda_{1}{£}_{L1}+\lambda_{2}{£}_{CE}+\lambda_{3}{£}_{\mathrm{AUX1}}+\lambda_{4}{£}_{\mathrm{AUX2}}+\lambda_{5}{£}_{DS}.$$ (7)

## 4 Experiment

### 4.1 Experimental setup

To validate the effectiveness of DifSeg for distance estimation of non-fixed obstacles, we evaluate PMOD-Net with DifSeg (PMOD-Dif) by using the following three datasets:

•Simulation dataset (CARLA (Dosovitskiy et al., 2017) dataset).
•Public outdoor dataset (KITTI-360 (Liao et al., 2022) dataset).
•Original indoor dataset.

We compare PMOD-Dif with PMOD-Net and NDDepth (Shuwei et al., 2024) on all datasets. On the KITTI-360 dataset, we can use the official NDDepth model without estimating scale because it is pre-trained by KITTI. On CARLA and original indoor datasets, we compare the performance by fine-tuning the pre-trained official NDDepth model on the datasets used for training PMOD-Net and PMOD-Dif.

In this experiment, the parameters of PMOD-Net for training are the same as in the original paper (Shikishima et al., 2023). Similarly, the parameters of PMOD-Dif for training that are used in Equation 7 are set as $\lambda_{1}=0.5$ , $\lambda_{1}=0.5$ , $\lambda_{2}=0.3$ , $\lambda_{3}=0.25$ , and $\lambda_{4}=5.0$ , which are the same as those of PMOD-Net training. The parameter $\lambda_{5}$ is set as $\lambda_{5}=5.0$ considering $\lambda_{4}$ because both $\lambda_{5}$ and $\lambda_{4}$ are parameters related to the depth image.

### 4.2 Dataset

The simulation dataset was created using the CARLA (Dosovitskiy et al., 2017) autonomous driving simulator. In this experiment, we utilized seven different maps provided by CARLA. To ensure the diversity of training and test data, pedestrians and vehicles were placed at random on each map as non-fixed obstacles. We prepared 1,000 images for training from each map, and all 7,000 images were used for training. For the test, we prepared 500 images from each map along different routes from the training data, and a total of 3,500 images were tested. That is, PMOD-Net and PMOD-Dif were tested on an unknown map that was not used in training. There is no self-localization error when PMOD-Net and PMOD-Dif project the 3D map to input depth images.

We utilized the KITTI-360 dataset for the public outdoor dataset, which is a large-scale in-vehicle sensor dataset collected in the outskirts of Karlsruhe, Germany. In this experiment, the input data consisted of left camera images and a 3D map. We used 1,000 images from each of the nine sequences provided by KITTI-360 for training. The test was conducted on each sequence, utilizing all data from each sequence provided by KITTI-360. In each test, PMOD-Net and PMOD-Dif were trained using 8,000 images from sequences different from those used in the test. That is, PMOD-Net and PMOD-Dif were tested on an unknown sequence (map) that was not used in training. The self-poses for projection utilize the IMU/GPS localization system with a self-localization data provided by KITTI-360 (Liao et al., 2022).

**FIGURE 4:**
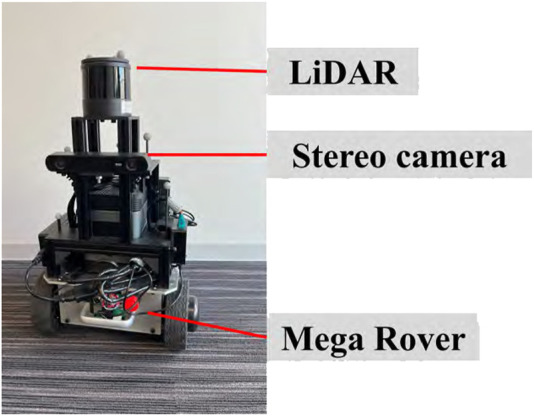
Mobile robot used for data collection of the original indoor dataset.

**FIGURE 5:**
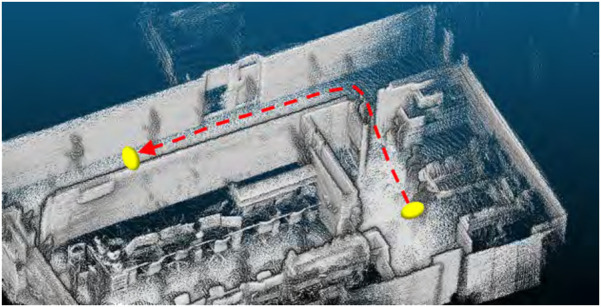
Route run by the mobile robot on the 3D map.

**FIGURE 6:**
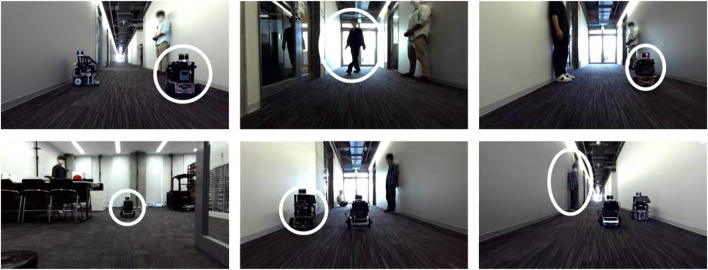
Examples of images in the original indoor dataset. The white circles shown in each image denote people and other robots, which are defined as non-fixed obstacles.

The original indoor dataset was collected by navigating in the Meijo University building using our mobile robot, as shown in Figure 4. A 3D map was created using point clouds obtained from the LiDAR attached to the top of the robot. Its model number is QT128. The test data were made from two round trips of our robot along the route shown in Figure 5 in the 3D map. In the training and test data, people and other robots crossed in front of our robot, or it followed them. We made four route datasets called “follow1,” “follow2,” “cross1,” and “cross2,” respectively. Figure 6 shows the appearance of people and other robots, used as non-fixed obstacles for training and testing in the original indoor dataset, which are indicated by white circles. The input camera images were collected by the left camera of the stereo camera attached to our robot. We fine-tuned the PMOD-Net and PMOD-Dif pre-trained on KITTI-360 with the training data collected from our robot. The number of images obtained for follow1, follow2, cross1, and cross2 are 62, 73, 57, and 74 frames, respectively. We perform 4-fold cross-validation with follow1, follow2, cross1, and cross2. GT segmentation images were made using LabelMe (Russell et al., 2008). GT depth images were created using CREStereo (Li et al., 2022) from stereo images collected from a stereo camera. The self-localization method performed with NDT matching (Biber and Straβer, 2003), which had a self-localization error of approximately 0.1 m. Note that even though our robot has a LiDAR and a stereo camera, we use just the left camera image and 3D map for the test. They are used to only get the data required by this experiment.

### 4.3 Evaluation index

We conducted the evaluation on non-fixed obstacles and fixed obstacles within the image. In the simulation and public outdoor dataset, pedestrians and vehicles were defined as non-fixed obstacles. In the original indoor dataset, people and other robots were defined as non-fixed obstacles. We defined fixed obstacles as those within the image that are not non-fixed obstacles. We utilized mean absolute error (MAE) and mean absolute percentage error (MAPE) for evaluation. These indicate the distance estimation error of obstacles in the image. MAE and MAPE are calculated using Equations 8, 9, respectively.

$$MAE=\frac{1}{n}\overset{n}{\underset{i=1}{\sum }}|D_{p}\left(i\right)-D_{GT}\left(i\right)|.$$ (8)

$$MAPE=\frac{1}{n}\overset{n}{\underset{i=1}{\sum }}\frac{\left|D_{p}\left(i\right)-D_{GT}\left(i\right)\right|}{D_{GT}\left(i\right)}.$$ (9)

### 4.4 Experimental results and discussion

**FIGURE 7:**
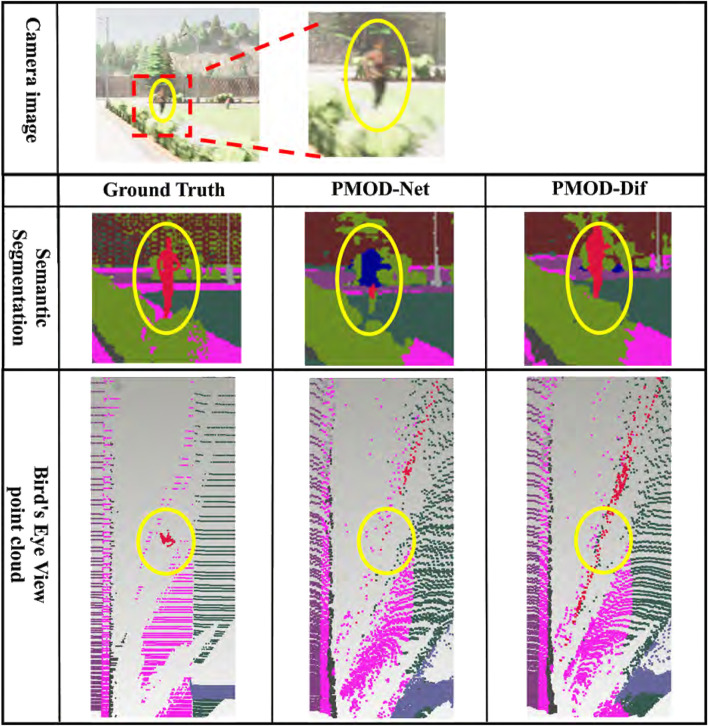
Estimation results in CARLA. From top to bottom: the camera image for input, the visualized segmentation images, and the bird’s eye view point clouds. Each bird’s eye view point cloud is a point cloud backprojected from the depth image and captured from a bird’s eye perspective. For visualization, it is colored using the ground truth segmentation image. The red point cloud represents a pedestrian.

**FIGURE 8:**
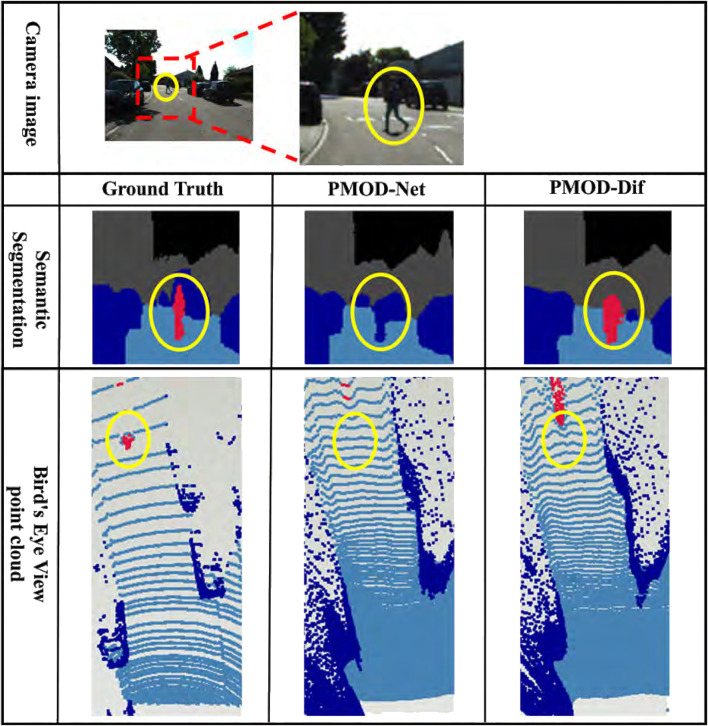
Estimation results in KITTI-360. From top to bottom: the camera image for input, the visualized segmentation images, and the bird’s eye view point clouds. Each bird’s eye view point cloud is a point cloud backprojected from the depth image and captured from a bird’s eye perspective. For visualization, it is colored using the ground truth segmentation image. The red point cloud represents a pedestrian.

**FIGURE 9:**
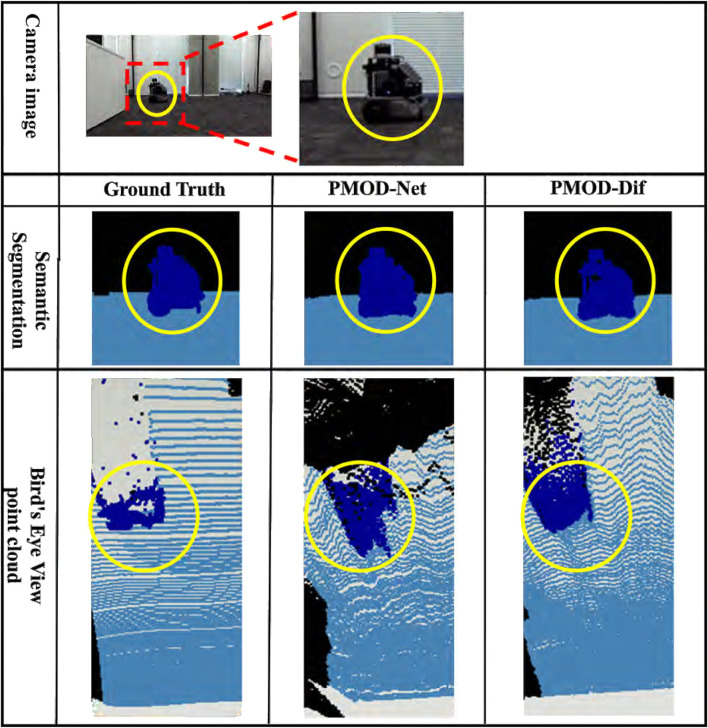
Estimation results in our original indoor dataset. From top to bottom: the camera image for input, the visualized segmentation images, and the bird’s eye view point clouds. Each bird’s eye view point cloud is a point cloud backprojected from the depth image and captured from a bird’s eye perspective. For visualization, it is colored using the ground truth segmentation image. The blue point cloud represents other robot.

The results for each dataset are shown in Tables 2–4. Figures 7–9 show the PMOD-Net and PMOD-Dif (ours) estimation results and the bird’s eye view point cloud created from the estimation results. We performed t-test on the original indoor dataset because its size is smaller than that of the CARLA and KITTI-360 datasets. The results of t-test showed that the differences of MAE and MAPE between PMOD-Dif and other methods were statistically significant (p < 0.01).

**TABLE 2:** MAE[m] and MAPE in CARLA.

Methods	Vehicles		Pedestrians		Fixed obstacles	
	MAE	MAPE	MAE	MAPE	MAE	MAPE
PMOD-Net	2.37	0.167	5.05	0.419	1.04	0.045
NDDepth	2.82	0.159	4.03	0.340	2.44	0.086
PMOD-Dif	1.84	0.129	3.57	0.319	1.04	0.046

**TABLE 3:** MAE[m] and MAPE in KITTI-360.

Methods	Vehicles		Pedestrians		Fixed obstacles	
	MAE	MAPE	MAE	MAPE	MAE	MAPE
PMOD-Net	2.49	0.236	4.55	0.429	1.05	0.087
NDDepth	4.56	0.346	5.27	0.370	3.50	0.305
PMOD-Dif	2.42	0.234	4.55	0.426	1.14	0.099

**TABLE 4:** MAE[m] and MAPE in the original indoor dataset.

Methods	Other robots		People		Fixed obstacles	
	MAE	MAPE	MAE	MAPE	MAE	MAPE
PMOD-Net	0.36	0.147	0.37	0.171	0.42	0.087
NDDepth	1.23	0.698	0.98	0.510	0.92	0.562
PMOD-Dif	0.33	0.109	0.27	0.133	0.41	0.069

From these results, the DifSeg loss improves the accuracy of distance estimation for non-fixed obstacles. Especially, Figures 7, 8 show that distant pedestrians, who could not be detected by conventional PMOD-Net, can be detected by PMOD-Dif.

Furthermore, as shown in Table 3, PMOD-Net provides higher distance estimation accuracy than NDDepth which is currently a highly accurate method on the KITTI benchmark. When restricted to vehicle class MAE and compared to NDDepth, the conventional PMOD-Net reduced the error by 2.07 m. Additionally, the adoption of DifSeg reduced the error by 2.14 m. These results indicate that the 3D map input is capable of estimating distance closer to the metric scale.

Comparing Tables 2, 4 with Table 3, the accuracy improvement rate on the KITTI-360 is low. We consider this is because there are fewer non-fixed obstacle regions in KITTI-360 than in our simulation and original indoor dataset. Therefore, we expect to improve accuracy by dynamically adjusting the weights of the DifSeg loss according to the size of non-fixed obstacle regions.

In this experiment, we found that PMOD-Net worked when the self-localization error was less than approximately 0.1 m. However, we do not obtain the self-pose on a 3D map by a camera in this experiment. The self-pose error of CMRNet (Cattaneo et al., 2016) that estimates self-pose on a 3D map by using monocular camera is 0.27 m. Therefore, we want to integrate PMOD-Net with a monocular camera self-localization method such as CMRNet in order to verify its relevance to the self-localization error.

For autonomous driving application, the inference time is important. Using the original indoor dataset, the mean inference times of PMOD-Net, NDDepth, and PMOD-Dif are 124 ms, 545 ms, and 113 ms, respectively. They are processed on our PC with a 12th Core i9 and a Geforce RTX 3060. PMOD-Net and PMOD-Dif are faster than NDDepth. However, for embedded systems, they are too slow. In future work, we have to make it faster by utilizing a fast neural network such as MobileNetV2 (Sandler et al., 2018).

For autonomous driving safety, the false detection of vehicles and pedestrians on the road is one of the big problems. Therefore, we also check the false positive F defined by Equation 10 on each dataset.

$$F=\frac{d}{s}.$$ (10)

d denotes the number of pixels where each method wrongly detects vehicles (other robots) and pedestrians (people) on the road pixels. s denotes a number of pixels that shows the road on the GT segmentation images.

Table 5 shows false positive of each dataset. Table 5 shows that all false positives are less than 1%, and the difference between PMOD-Net and PMOD-Dif is small. We think that DifSeg does not affect the false positive very much because it is based on the error of depth, as shown in Equation 2.

**TABLE 5:** False positive [%].

Methods	CARLA	KITTI-360	Original indoor
PMOD-Net	0.17	0.36	0.30
PMOD-Dif	0.17	0.38	0.21

## 5 Conclusion

This study addressed the challenge of improving the accuracy of distance estimation for non-fixed obstacles that do not exist on a 3D map. We focused on PMOD-Net that simultaneously output semantic segmentation image and depth image by using 3D map. During the training of PMOD-Net, we add a new loss “DifSeg” that reduces the difference of distance on the non-fixed obstacle region obtained from a segmentation image. DifSeg realizes training focusing on the detected non-fixed obstacle region. The accuracy of distance estimation was improved on the CARLA, KITTI-360, and original indoor datasets. Specially, on the KITTI-360, the distance estimation error of our method was 2.42 m, which was 2.14 m less than that of the latest monocular depth estimation method. Moreover, the capability of the 3D map was also clarified. Future work will include dynamic changes of the weights of the DifSeg loss depending on the size of the non-fixed obstacle region.

## Data Availability

The raw data supporting the conclusions of this article will be made available by the authors, without undue reservation.
